# NiO-Based Aerogels—Unexpected Formation of Metallic Nickel Nanoparticles during Supercritical Drying Process

**DOI:** 10.3390/nano12224033

**Published:** 2022-11-17

**Authors:** Elena A. Straumal, Andrey A. Mazilkin, Inna O. Gozhikova, Lyudmila L. Yurkova, Sergey Yu. Kottsov, Sergey A. Lermontov

**Affiliations:** 1 Institute of Physiologically Active Compounds at Federal Research Center of Problems of Chemical Physics and Medicinal Chemistry, Russian Academy of Sciences, 1 Severnij Pr., Chernogolovka, 142432 Moscow, Russia; 2 Institute of Solid State Physics, Russian Academy of Sciences, 2 Academician Ossipyan Str., Chernogolovka, 142432 Moscow, Russia; 3 Kurnakov Institute of General and Inorganic Chemistry, Russian Academy of Sciences, 31 Leninsky Prosp., 119991 Moscow, Russia

**Keywords:** aerogels, supercritical drying, sol–gel synthesis, nickel oxide, metallic nickel nanoparticles

## Abstract

The aim of the study is to investigate the influence of the solvents applied both in sol–gel process and for supercritical drying (SCD) on NiO aerogels’ properties. NiO aerogels were synthesized using methanol and 2-methoxy-ethanol (MeGl) as sol solvents. SCD was performed using iso-propanol, methanol and tert-butyl-methyl ether as supercritical fluids. The obtained samples were characterized using low-temperature nitrogen adsorption, X-ray diffraction analysis, mass-spectra analysis and STEM and TEM methods. It was found that specific surface area and the phase and chemical composition strongly depend on the synthesis conditions. We revealed that Ni^2+^ cations were reduced into Ni^0^ when 2-methoxy-ethanol was applied as a sol solvent. The mechanism of the Ni^2+^ cations reduction is proposed. We consider that at the stage of sol preparation, the Ni^2+^–MeGl chelate was formed. This chelate decomposes at the SCD stage with the release of MeGl, which, in turn, eliminates methanol and leads to the formation of aldehyde. The latter is responsible for the nickel reduction. The proposed mechanism was confirmed experimentally.

## 1. Introduction

Ni(II) oxide is of considerable interest due to its unique properties. NiO is known to be a semiconductor and it is also used as a catalyst for several chemical transformations including the reforming of methane by CO_2_ [1,2] and the oxidation of H_2_S to sulfur [3]. The properties of NiO materials directly depend on such parameters as particle size and morphology, crystallinity and specific surface area. The synthesis method used can significantly vary these parameters [4,5,6].

Sol–gel synthesis is a method that allows obtaining materials with enhanced purity, homogeneity and tunable textural features (porosity, specific surface area, particle size) [7]. Aerogels are unique materials with uncommon properties (low density, high porosity, and large specific surface area, etc.) making them attractive for several applications, namely for thermal and acoustical insulation [8,9,10], as catalysts and catalysts support [11,12,13,14], as sorbents [11,12] and so on. They are usually obtained by a multistage method. The first stage is a sol–gel synthesis of so-called lyogels, and the last stage is transforming the lyogel into aerogel by a supercritical drying method. In our previous works, we have demonstrated that the nature of supercritical fluid significantly affects the parameters of the synthesized aerogel: the values of specific surface area, crystallinity, phase and chemical composition of aerogels strongly depend on supercritical fluid nature [15,16,17].

Numerous articles are focused on the synthesis and study of binary or ternary aerogels of NiO based on silica and alumina [18,19,20,21,22,23,24,25]. However, there are only a few studies on obtaining and investigating individual NiO aerogels [6,26]. Gash et al. [6] described an epoxide-assisted method for synthesizing nickel oxide aerogels. Ethanol was used as a sol solvent and nickel (II) chloride and nitrate were used as metal precursors. The authors revealed that monolithic NiO aerogel was formed from a NiCl_2_ × 6H_2_O ethanolic solution, while only a precipitate (not a gel) was formed from a Ni(NO_3_)_2_ × 6H_2_O solution. Another synthesis procedure was implemented by Zhang et al. [26]. NiO aerogels were prepared by a citric acid-assisted sol–gel method from an ethanolic solution of Ni(NO_3_)_2_ × 6H_2_O.

In this work, we investigate the influence of the solvents applied both in sol–gel process and for supercritical drying on NiO aerogels’ properties: values of specific surface area, phase composition and microstructure.

## 2. Experimental Section

### 2.1. NiO-Based Aerogels Preparation

Nickel (II) chloride hexahydrate NiCl_2_ × 6H_2_O (Aldrich, 99.9%), propylene oxide (Aldrich, Belgium 99%), isopropyl alcohol (Aldrich, Belgium 99.5%), methyl-*tert*-butyl ether (MTBE) (Aldrich, Belgium 99%) and 2-methoxyethanol (MeGl, methylglycol) (Aldrich, Belgium 99.8%) were used in the as-received state. Sols were prepared according to the method described in [6]. Namely, 0.37 g NiCl_2_ × 6H_2_O (1.56 mmol) was dissolved in 2.5 mL of methanol or methoxyethanol (MeGl) to give a clear light green solution. Then propylene oxide (1 g, 17 mmol) was added under continuous stirring.

The obtained sols were transferred to plastic molds covered by Parafilm, and the solutions were allowed to gel at room temperature. Gel formation typically occurred within 10–15 min for methanol and approximately 60 min for MeGl. The obtained samples were washed by soaking five times using isopropyl alcohol, MTBE or MeOH. The solvent was exchanged daily with a fresh portion. After being washed, the aerogels were dried under supercritical conditions in the same solvents.

Supercritical drying (SCD) was performed as follows. Lyogel (gel before drying) samples were put into a stainless steel autoclave (V ≈ 40 mL). The autoclave was filled with a solvent (V ≈ 20 mL) and heated. The system was heated to a temperature of 15–20 °C above the solvent critical point. The heating rate was approximately 50 °C/h. The critical point is 235 °C for i-PrOH, 239 °C for MeOH and 224 °C for MTBE. When the required temperature was reached (the pressure in the autoclave was 20–22 MPa for all solvents), the valve was opened and the pressure decreased down to the ambient value in 2 h. The autoclave was then evacuated in 20–30 min (2–5 mmHg), cooled to room temperature and then opened.

### 2.2. Investigation Methods

The specific surface area (SSA) of the aerogels was determined by measuring low-temperature nitrogen adsorption with an ATX–06 analyzer using the 5-point BET method. X-ray diffraction (XRD) analysis was carried out on a SIEMENS D-500 diffractometer (CuKα_1_ radiation). Infrared (IR) spectroscopy was performed on a Bruker IFS-113V spectrometer in a 4000–350 cm^–1^ wavenumber range. The identification of gaseous products evolved during the thermal decomposition of the samples was performed using a NETZSCH QMS 403 C Aëolos quadrupole mass spectrometer. The analysis was carried out in Ar at a heating rate of 10 °C/min to 800 °C. Scanning electron microscopy was performed on a Versa 3D dual-beam instrument with an acceleration voltage of 30 kV and a STEM II HAADF detector. Transmission electron microscopy (TEM) was carried out on a JEM 2100 microscope at 200 kV.

## 3. Results and Discussion

In this paper, we use the following notation: MeOH sols (MeOH samples) and MeGl sols (MeGl samples) are sols and aerogels prepared using MeOH and methoxyethanol (MeGl) as sol solvents, respectively. The lyogels obtained from MeOH sol are bright green and transparent, while the lyogels obtained from MeGl sol are light green and light-tight.

The specific surface area of the aerogel samples depending on sol and SCD solvents is provided in Table 1.

It is clearly seen that SSA value directly depends on the solvents used for sol preparation and for SCD. The nature of solvents’ influence on the gel textural properties is a well-known phenomenon [27]. In our previous studies, we have shown as well that both the supercritical drying (SCD) solvent [15,16,17] and sol solvents [28] affect the specific surface area of aerogels significantly.

**Figure 1:**
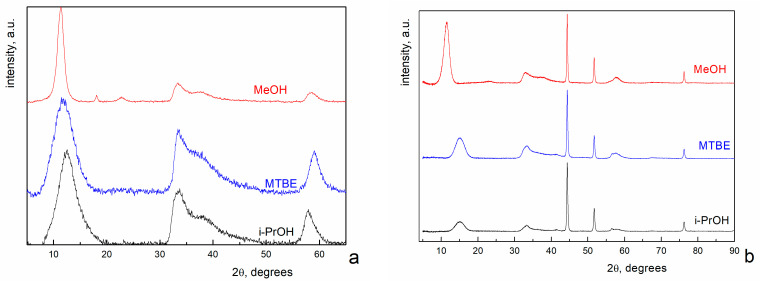
X-ray diffraction patterns of i-PrOH, MTBE and MeOH NiO aerogels prepared from MeOH sol (**a**) and MeGl sol (**b**).

Figure 1a shows the XRD pattern of i-PrOH, MTBE and MeOH NiO aerogels, prepared from MeOH sols. XRD data demonstrate similar phase composition of all three samples. The diffraction profiles can be fit with either Ni(OH)_2_ × 2H_2_O (JCPDS #22-444), or Ni_3_(OH)_4_(OCH_3_)_2_ (JCPDS #30-1835), or their mixture. Actually, there are two peaks in the diffraction pattern of the sample dried in MeOH that are absent in the other two patterns (Figure 1a). One of them (at 2θ~23 degrees) can be attributed to Ni(OH)_2_ × 2H_2_O (JCPDS #22-444), Ni_3_(OH)_4_(OCH_3_)_2_ (JCPDS #30-1835) or their mixture. It is well known that methanol has an increased dissolving ability. For example, the solubility of SiO_2_ in methanol is by an order of magnitude higher than that in other alcohols [29]. Based on this fact, the solubility of Ni oxides in methanol is expected to be higher than that in i-PrOH or MTBE. We believe that the increased solubility in methanol makes it possible to form a more distinct crystalline structure, and we can observe even low-intensity peaks on the diffractogram.

The second peak (at 2θ~19 degrees) cannot be attributed to the suggested phases. Taking into account the elemental composition of the sample, it can belong to the NiOOH phase (JCPDS #6-141).

Summarizing the considerations above, we can conclude that the main phase in samples obtained from MeOH sols is Ni(OH)_2_ × 2H_2_O (JCPDS #22-444), Ni_3_(OH)_4_(OCH_3_)_2_ (JCPDS #30-1835) or their mixture. XRD analysis does not allow us to choose between these three variants.

**Figure 2:**
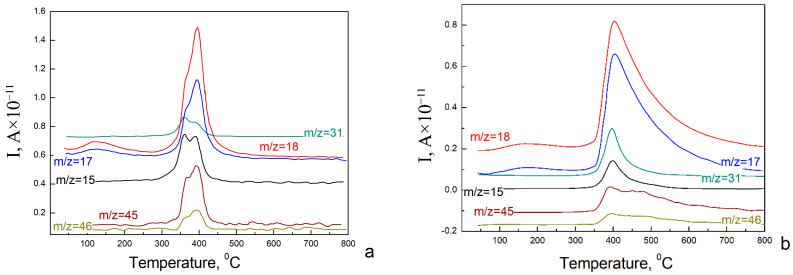
Mass spectra of NiO aerogels obtained from MeOH sol and dried under supercritical conditions in i-PrOH (**a**) and MeOH (**b**).

Additional information about the chemical and consequently phase composition of the samples was provided by means of mass spectroscopy. The samples were heated in the spectrometer chamber, and the products of the pyrolysis process were analyzed. The obtained mass spectra are shown in Figure 2.

Peaks on these spectra indicate the appearance of ionic current corresponding to the following compounds: CH_3_O (31), H_2_O (18), OH (17), CH_3_ (15), CH_3_OCH_3_ (46) and CH_3_OCH_2_ (45). Mass spectra analysis allows us to conclude that the aerogels surfaces are covered by hydroxyl and methoxy groups. The appearance of methoxy groups on the surface can be explained by using methanol to prepare the initial sol. The elimination of these groups occurs at a temperature above 300 °C. This means that these groups are covalently bound to the aerogel surface.

Comparing the data obtained by mass spectroscopy and XRD, we can conclude that all NiO samples obtained from MeOH sol are a mixture of Ni_3_(OH)_4_(OCH_3_)_2_ and Ni(OH)_2_ × 2H_2_O.

Figure 1b shows XRD patterns of i-PrOH, MTBE and MeOH NiO aerogels prepared by supercritical drying from MeGl sols. The profiles contain two different sets of diffraction peaks. Narrow peaks with high intensity at 2θ = 44.5°, 51.8° and 76.4° correspond to Ni^0^ (JCPDS #4-850). The remaining peaks are exactly the same as in Figure 1a. It should be noted that these peaks correspond to Ni(OH)_2_ × 2H_2_O in the case of MeGl samples since the formation of methoxide is impossible in the absence of methanol. In other words, the use of MeGl for sol preparation leads to the formation of the Ni^0^ phase in the aerogel samples.

The full width at half maximum for the Ni^0^ peaks is about 0.2°, which is comparable to the value of the standard sample. This means that it is not correct to estimate the crystallite size by the peak broadening. We performed SEM and TEM studies to have more insight into the sample structure.

**Figure 3:**
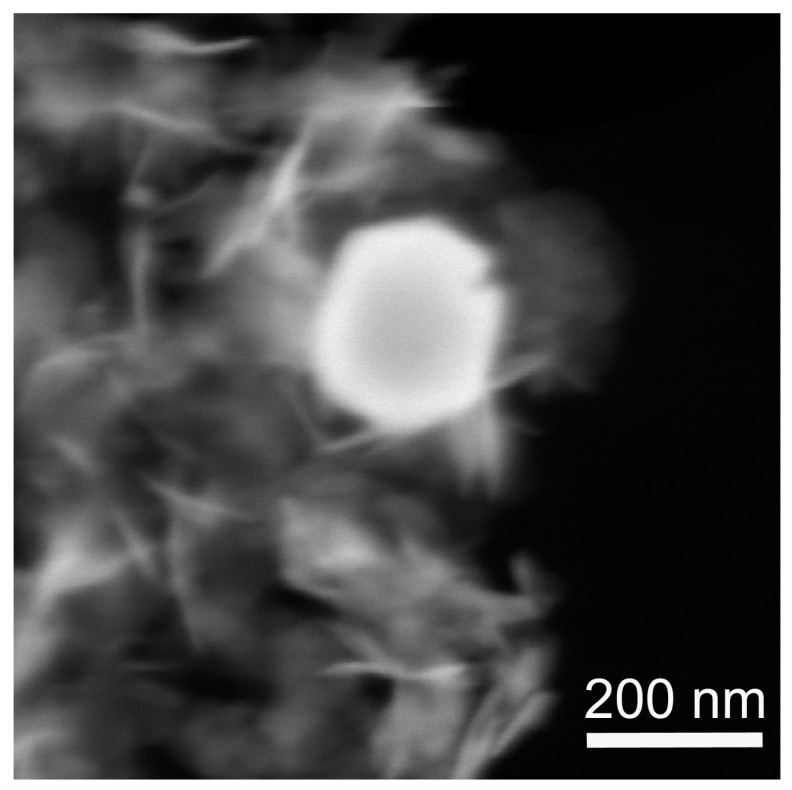
SEM image of the NiO aerogel obtained from MeGl sol and dried under supercritical conditions in i-PrOH. The image was acquired in transmission mode with a HAADF detector.

Figure 3 demonstrates the microstructure of the sample obtained from MeGl sol and dried under supercritical conditions in i-PrOH. The sample structure contains two constituents, i.e., comparatively large well-faceted particles, and flakes with irregular shapes. Big particles show a brighter contrast in the HAADF STEM image and seemingly belong to the Ni^0^ phase, having a higher average atomic number. To confirm this conclusion, we performed a TEM study of the same sample.

**Figure 4:**
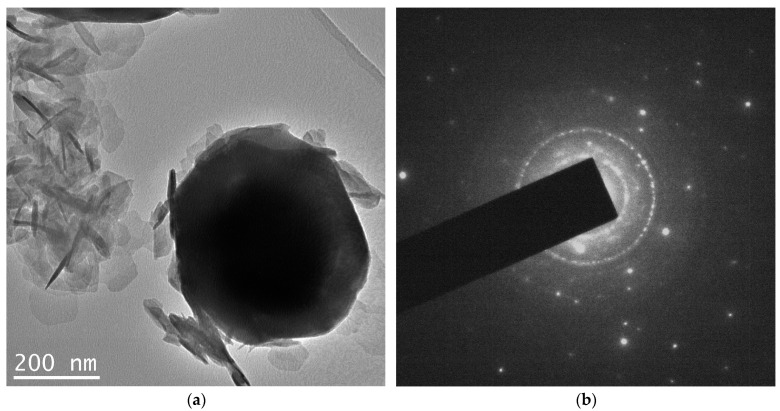

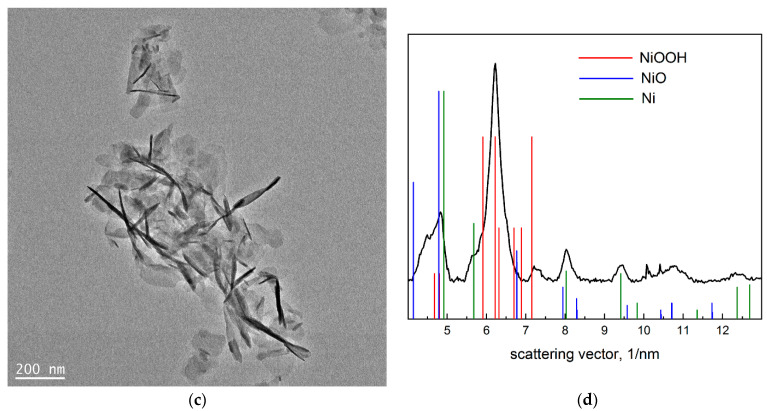
TEM data on the NiO aerogel obtained from MeGl sol and dried under supercritical conditions in i-PrOH: (**a**) micrograph of Ni0 particle and oxide flakes; (**b**) diffraction pattern of the area in (**a**); (**c**) micrograph of oxide flakes; and (**d**) annular intensity distribution of diffraction pattern in (**b**).

The TEM results can be seen in Figure 4. Here we see again large faceted particles along with a cloud-like cluster of flakes. The selected area diffraction pattern (DP) shows the presence of sharp individual reflections, as well as those giving the circle diffraction pattern. The analysis of the DP was performed based on the annular intensity distribution (Figure 4c). The bar diagram in this figure shows the reflection positions for three phases, i.e., NiOOH, NiO and metallic nickel.

According to the XRD data (Figure 1b), samples obtained from MeGl sol consist of a mixture of two phases—Ni(OH)_2_ × 2H_2_O and Ni^0^. The presence of metallic nickel is also confirmed by electron diffraction data (Figure 4d). As for the second phase in TEM images (flakes), DP data give a structure different from that in XRD. According to electron diffraction data, these flakes can consist of NiOOH and NiO phases, whereas XRD gives Ni(OH)_2_ × 2H_2_O, except for Ni. The reason for the difference in phase composition can be explained by the beam damage, which occurs during beam–sample interaction. The most significant mechanism of beam damage for the samples under the study is radiolysis [30]. As a result of the radiolysis, Ni(OH)_2_ × 2H_2_O loses both crystallization water and part of the chemically bonded hydroxyl groups, which leads to the formation of NiOOH and NiO phases. However, the main result from the TEM study is the assessment of the Ni grain size, which was not possible from the XRD due to the fact that it is quite large. The average particle size calculated from TEM data was approximately 490 nm for metallic nickel and 75 nm for oxide flakes.

Several experimental studies are available in the literature on obtaining Ni^0^ by the thermal decomposition of nickel carboxylates in an inert atmosphere [31,32,33,34] or by the reduction of nickel hydroxide by heating in a hydrogen atmosphere [35].

Ni^2+^ reduction under the condition described above was rather surprising, and we tried to find an explanation for it.

**Figure 5:**
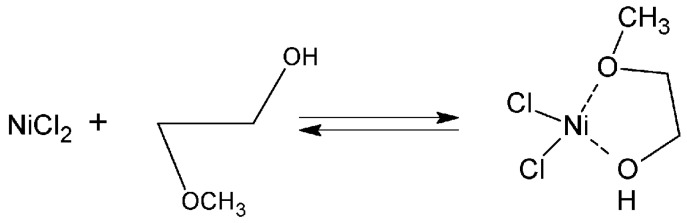
Scheme of chelate formation.

The MeGl molecule has two donor oxygen atoms. Therefore, it can efficiently chelate metal ions with the formation of a stable five-membered cycle according to Figure 5 [36]. A similar phenomenon was observed for nickel ions in [37].

**Figure 6:**
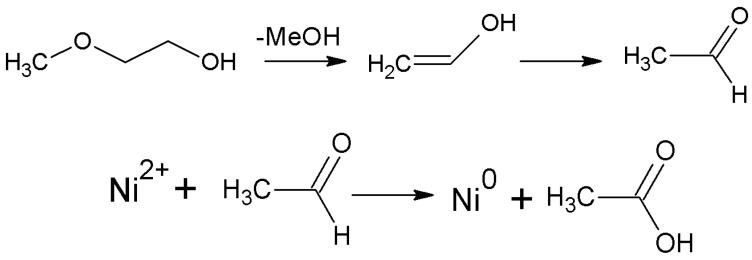
Scheme of aldehyde formation from MeGl and Ni^2+^ reduction in the presence of aldehyde.

This reaction is reversible, and chelates can decompose in the process of SCD releasing MeGl. MeGl can produce aldehyde under the action of a Lewis acid Ni^2+^ (according to scheme in Figure 6) which subsequently can reduce nickel ions according to the scheme given in Figure 6.

To confirm this assumption, we performed the following additional experiments: First, to accurately reproduce the SCD drying conditions, the MeGl was heated in an autoclave in the presence of NiCl_2_ at 250 °C (the temperature of SCD). The obtained reaction mixture was examined by IR spectroscopy. The absorption band at 1730 cm^–1^ indicates the presence of the aldehyde carbonyl group in the obtained mixture. It allows us to conclude that the heating of MeGl in the presence of Ni(II) chloride leads to the formation of acetaldehyde.

Second, to confirm that aldehyde alone is responsible for Ni^2+^ reduction, we subjected the MeOH lyogel to SCD in i-PrOH in the presence of propionaldehyde (acetaldehyde is carcinogenic and too volatile—b.p. at 20 °C). We found that the obtained aerogel contained metallic nickel. It is known that aliphatic aldehydes have tautomeric enol-form. So, we can suppose that not aldehyde but –C=C– double bond can act as a reducing agent. To understand which of these groups is responsible for Ni^2+^ reduction, we repeated the experiment using p-methyl-benzaldehyde which has no enol form due to its chemical structure. It was found that even in this case the samples contained Ni^0^. As mentioned above, no Ni^0^ was found after the SCD of MeOH lyogels without aldehyde (see Figure 1a). Therefore, we can conclude that the presence of aldehyde is the cause of Ni^2+^ to Ni^0^ reduction.

## 4. Conclusions

NiO-based aerogels were synthesized by the epoxide-assisted sol–gel method. Methanol and 2-methxyethanol were used for sol preparation. Methanol, iso-propanol and tert-butyl-methyl-ether were used for supercritical drying. It was shown that value of the specific surface area depends on both the sol solvent and the SCD solvent. The study revealed that samples obtained from MeOH sol consisted of Ni_3_(OH)_4_(OCH_3_)_2_ but can also contain Ni(OH)_2_ × 2H_2_O. Samples obtained from MeGl sol consisted of Ni(OH)_2_ × 2H_2_O and metallic nickel. Ni^2+^ reduction was caused by the formation of aldehyde from 2-methoxy-ethanol in presence of Ni^2+^ ions.

## Figures and Tables

**Table 1:** The SSA values (m^2^/g) of NiO aerogel samples.

	SCD Solvent		
	i-PrOH	MTBE	MeOH
MeOH sol	336	405	139
MeGl sol	650	430	178

## Data Availability

The data is available on reasonable request from the corresponding author.
